# Validation of a Unique Boxing Monitoring System

**DOI:** 10.3390/s21216947

**Published:** 2021-10-20

**Authors:** Tobias Menzel, Wolfgang Potthast

**Affiliations:** Institute of Biomechanics and Orthopaedics, German Sport University Cologne, 50933 Cologne, Germany; potthast@dshs-koeln.de

**Keywords:** instrumented sports equipment, boxing monitoring system, Smart Wearable, punch force, strike trajectory, biomechanics analysis

## Abstract

Much development work and scientific research has been conducted in recent years in the field of detecting human activity and the measurement of biomechanical performance parameters using portable sensor technologies, so-called wearable systems. Despite the fact that boxers participating in one of the most vigorous and complex disciplines of all sports, it is one of the disciplines where no noteworthy, advanced performance analytic tools are used for training or for competition purposes worldwide. This research aimed to develop and validate a comprehensive punch performance sensor system for the measurement and analysis of biomechanical parameters in the sport of boxing. A comprehensive validation study on linear regression was conducted following the development of the sensor system, between the gold standard of a Kistler force plate and Vicon motion capture system, to compare sensor-derived measurements with the gold standard-derived measurements. The developed sensor system demonstrated high accuracies ranging from R^2^ = 0.97 to R^2^ = 0.99 for punch force, acceleration, velocity and punch-time data. The validation experiments conducted demonstrated the significant accuracy of the sensor-derived measurements for predicting boxing-specific biomechanical movement parameters while punching in field use. Thus, this paper presents a unique sensor system for comprehensive measurements of biomechanical parameters using the developed mobile measurement system in the field of combat sports

## 1. Introduction

With increasing professionalism and popularity of a sport, the equipment used also develops in terms of specialization [1]. Significant development work and scientific research has been conducted in recent years in the field of detecting human activity and the measurement of biomechanical performance parameters using portable sensor technologies, so called ‘wearables’ [2,3].

Despite this sustained focus, users remain largely unaware of the extent to which the data provided by wearable sensor technologies are reliable and accurate [4]. The lack of information about the validity in terms of reliability and accuracy of developed wearable sensor technologies as well as the need for their validation to evaluate the effectiveness of the sensors for the athletes has been discussed by many authors as a crucial part of the development process [2,3,5,6,7,8,9].

Compared to other disciplines, the sport of boxing is exceptional in regard to the evolution of equipment used and the technical instruments applied for performance measurement and in-field monitoring.

It must be noted that historically, various instruments have been developed and used to determine the force and speed of different punching techniques, such as the ‘cross’, ‘jab’, ‘hook’ or ‘uppercut’, as the most popular punches thrown during a boxing match [10,11,12,13,14,15,16]. Although punching accuracy and speed are important performance-determining parameters in boxing matches, several studies have shown that punching force is the main performance indicator for success or failure in boxing matches [13,17,18,19]. In professional boxing and heavyweight fights particularly, single hard hits can lead to a knock-out, and thus, to victory.

Previous studies have used various instruments to measure impact forces under laboratory conditions. An overview of these different measuring techniques is presented in Table 1.

As stated above, punch force is a critical factor in evaluating the efficacy of training programs. Athletes and coaches currently lack any measuring instruments to analyze the force of impact, punch speed or other important biomechanical parameters in training or competition with a high degree of accuracy.

To address this gap, we have developed a unique, comprehensive boxing performance monitoring system, which uses a force-sensing resistor, based on the piezoresistive principle, as well as a combination of accelerometer, gyroscope and magnetic sensors. The developed system can be incorporated in a boxing glove without significant manipulations of the glove’s physical properties.

Crucial to the development of the sensor system is the validation of the sensor data, with existing measurement systems. Therefore, the aim of this research was to validate the developed sensor system. Due to the parameters generated, a Kistler force plate and Vicon motion capture system were used in this validation study, which are considered gold standard [4,24,25] to test linear regression.

## 2. Materials and Methods

### 2.1. Empirical Setup and Protocol

The validation process of the developed sensor system is divided into two parts.

The first part serves to validate the ‘impact force’ measurement, by using developed piezoresistive pressure sensors. Therefore, the force (N), measured by the sensor system, was validated against the force (N), measured by the Kistler force plate, which is the gold standard for the measurement of force [25]. To validate the accuracy of the measurement, impact tests were performed in such a way as to simulate the actual field of application.

The second part serves to validate the inertial sensor technology implemented within the glove. The large dynamic movements of fists during a boxing match require a special measuring method, because the gold standard motion capture system requires marker-based motion tracking with the help of camera systems to produce accurate measurements (VICON MXF40, Vicon Motion Systems Ltd., Oxford, UK). This method is not practical as it involves a high risk of injury and high probability of losing markers during the match. Therefore, the developed sensor system measures the trajectory and movement of the fist by incorporating calibrated inertial sensors. In addition, the sensor behavior was analyzed for long-term applications.

**Figure 1:**
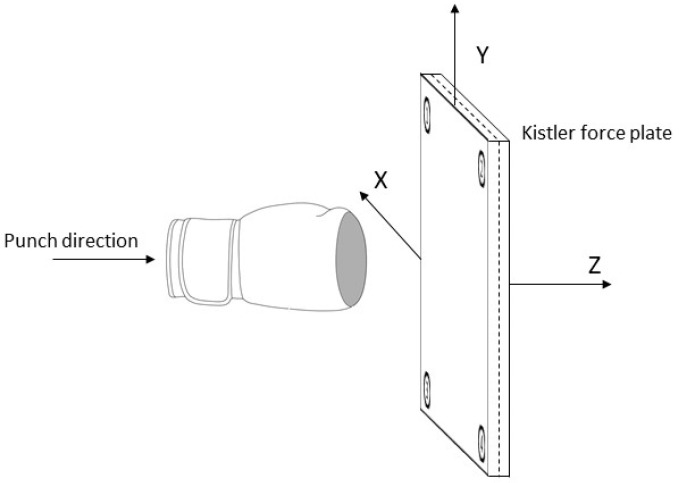
Test setup schematic.

The measurement setup of this study included the verification of the impact force with the use of a Kistler force plate, which, according to Roell et al., (2019), is the gold standard in the evaluation of the force measurement in biomechanical data systems. To validate the impact force, impacts were applied by straight punches to a Kistler force plate (Figure 1). The sensor system as well as the microelectronics used for the validation were integrated into a 12-ounce (340.194 g) AIBA certified Adidas boxing glove (2017 model). A sampling frequency of 1000 Hz was used to measure the acting impact forces. The data acquisition of the Kistler force plate was conducted with a frequency of 10,000 Hz. The Kistler data processing was performed using Vicon Nexus software for motion capture in life sciences.

The measurement protocol consisted of four validation runs which were conducted on two consecutive days. For each of the four measurement runs, fifteen impact sequences were performed on the designated Kistler force plate. The cross punch was determined as the most repeatable punching technique and, consequently, selected for the experiment. In order to validate the most extensive range of impact force possible, as it is to be expected in field, impact forces with a range from 200 N to 2300 N were executed.

The second part of the study examines the measurement validity of the inertial sensors used. In order to validate the entire potential measuring range of ±200 g, a special validation device was developed based on the principle of a centrifugal device. Based on preliminary tests, the sensor range of ±200 g was evaluated as sufficient. The maximum acceleration at impact was measured up to 160 g for internationally experienced athletes.

**Figure 2:**
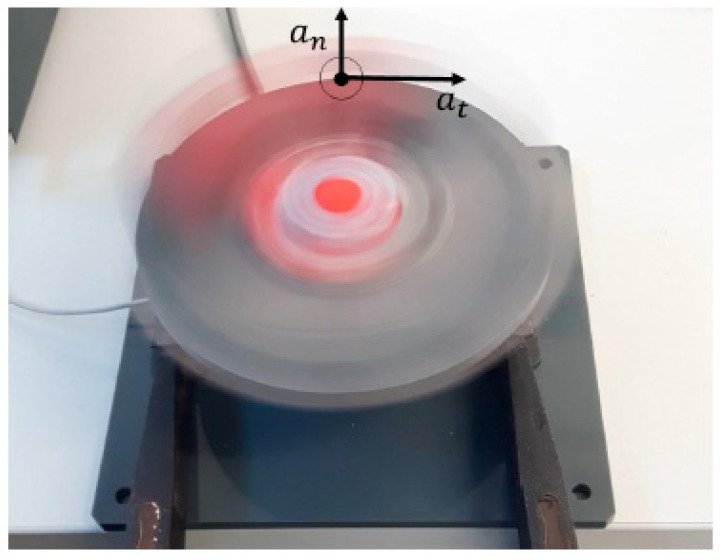
Centrifugal measurement data acquisition.

The constructed and designed centrifugal device is presented by Figure 2. The materials used were carefully selected to ensure that the centrifuge did not produce hard and soft iron effects that could falsify the extraction of the gravitational acceleration within the inertial measurement unit. In addition, the design was a lightweight construction to avoid additional load on the motor by the weight of the rotating disc. The materials used to manufacture the validation device was mainly extruded dark gray PVC-U plastic. In addition, drawn aluminum (AlCuMgPb) and rolled aluminum (AlZnMgCu1.5) were used for the mounting of the motor and the ball bearing drive shaft of the motor.

The mounting of the centrifuge and the motor was fixed on a plastic PVC-U base plate. A VEXTA^®^ Brushless DC Motor (AXH015K-A) from Oriental Motor’s USA Co. Ltd. (Torrance, CA, USA) drove the centrifuge plate from the base plate via a ball-bearing shaft. The ball-bearing shaft enabled the transmission of the rotational movement of the motor to the sensor platform without interference, even at high revolutions per minute (RPM). This design construction enabled the acceleration generation without stressing the engine shaft and causing damages to the motor or a deterioration of the exact rotational transmission. The entire design of the turntable was made from extruded dark gray PVC-U plastic to reduce the overall weight and mass moment of inertia of the turntable, which must be driven by the motor. The rotation plate consisted of a rotation disk that allowed the sensor to be mounted on top. During the design of the sensor system on the centrifuge plate, it was ensured that the deviation of the sensor would maintain the same distance from the center of rotation in all axes by using a mounting template to which the sensor was attached.

The design of the validation device enabled the adjustment of the input acceleration needed in the form of a rotational movement created by an electric motor. The concept of the validation device consisted of a fixed motor that accelerated a turntable and the sensor unit mounted on top via a ball-bearing shaft.

The experimental protocol of the acceleration validation by the developed centrifugal device consisted of 20 measuring cycles in each of the experimental runs. The measurement was from −200 g up to +200 g with a predefined increase of the acceleration rates for all three axes. Each of the measurement levels was held for a data acquisition period of a minimum of 50 s to collect a considerable number of data samples before accelerating to the next measurement level. This measurement protocol was repeated five times to analyze the system’s repeatability.

Following the acceleration validation process, the sensor system was validated through the orientation of the sensor in a three-dimensional space. This process was carried out in a first step by a gimbal device equipped with analogue goniometers for all three axes. The designed validation apparatus rotated the sensor around all three axes (yaw, pitch and roll) 360° without affecting the sensor positioning on the inner sensor mount.

The gimbal device was constructed and designed using the same material as was used for the centrifugal device. Therefore, extruded dark gray PVC-U plastic was used to avoid hard and soft iron effects that could interfere the sensor output for the determination of the angular rotation.

Potentiometers were used to generate a reference value to validate the rotation angle of the sensor around the respective axes against the rotation determined by the electrical signal of the potentiometer.

**Figure 3:**
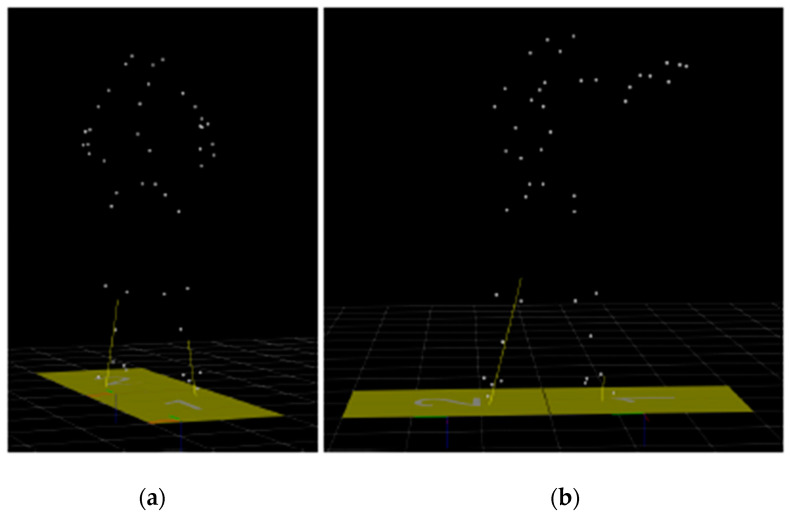
Vicon motion capture testing: (**a**) preparation phase, (**b**) throwing phase.

In the following stage, an extensive validation for the angular rotation, acceleration and velocity was performed by conducting boxing exercises in a motion analysis laboratory. In the experimental setup, punches against a boxing bag with a defined weight were performed. A 40 kg punching bag made out of leather from Paffen Sport (Paffen Sport GmbH & Co. KG, Cologne, Germany) was used on a wall-mounted suspension to perform the punches against a defined and stationary target. The three-dimensional movement execution of the glove was recorded utilizing a marker-based Vicon motion capture camera system as presented in Figure 3.

Drift tests were conducted in a subsequent step following the results of the punch force, angle and acceleration validation experiments. The drift tests were used to validate the system on drift occurring over time in action. Thereafter, the system was tested over a period of five, fifteen and finally forty-five min, according to the maximum length of a boxing match.

### 2.2. Data Analysis

To compare the acquired punch force validation data of the Kistler force plate with the sensor device, the sensor data had to be interpolated to be able to perform a holistic analysis of the force–time curve between both measurement systems, due to different data acquisition frequencies between the developed sensor system with a frequency of 1000 Hz, the force plate frequency of 10,000 Hz and the Vicon Motion Capture System with a frequency of 1.000 Hz. The data processing and further data analysis were performed using custom-built MATLAB (2018b) routines (The MathWorks, Natick, MA, USA) for all tests conducted during the experimental validation study.

The data analysis to validate the incorporated inertial sensors was performed in an identical manner. Due to the differences in recording frequencies, the sensor data were matched by means of data interpolation.

For the validation of the entire range of acceleration using the centrifugal device, the sensor axis to be validated (*an*) was aligned in the direction of the centrifugal force, orthogonally to the direction of the tangential acceleration (*at*) as shown in Figure 2. Whereas the orientation of the coordinate system for the validation of the orientation in a three-dimensional space using the developed angular rotation validation device was aligned with the *x*-axis pointing upwards, the *y*-axis pointing sideways and the *z*-axis pointing forward. This initial position was chosen as it is similar to the starting position of the boxing glove while the fist remains in the defensive position before it is tilted in a forward direction by 45° around the *y*-axis in the direction of the object to be hit, thus, assuming the punching orientation.

### 2.3. Statistical Analysis

The statistical data analysis of the individual validation experiments presented followed the preliminary data processing and was performed using the analysis software, IBM SPSS Statistics for Windows, Version 23.0 (IBM Corporation, New York, NY, USA).

To analyze the validity of the predicted punch force determination using the developed sensor system, a linear regression was performed based on the punch force data from the Kistler force plate. Linear regression analysis was also performed to validate the acceleration in g’s and angular rotation in degrees, as determined by the inertial sensor. A Pearson correlation coefficient analysis was conducted to analyze the sensor systems validity. For the visualization of the statistical analysis, a scatter plot is used to show linear correlation between sensor-derived punch force (N) and Kistler force plate-derived punch force (N). Scatter plots were used to show linear regression between inertial sensor-determined acceleration (g) and centrifugal-derived acceleration (g) as well as inertial sensor-determined rotation (°) versus potentiometer-derived rotation (°). The same statistical analysis method was conducted to test the incorporated inertial sensor unit in the second step against the Vicon motion capture system. In addition to the Pearson correlation coefficient, the root means square error (RMSE) as well as the standard deviation (SD) was calculated to evaluate the statistical data from the experimental validation results to provide detailed information about the magnitude of measurement errors.

To analyze the significance of the measurement results, an ANOVA statistic was carried out, to test on the statistical analysis of statistically significance with a selected alpha level of 0.05.

The data were further analyzed on the homoscedasticity of the residuals. A Durbin–Watson statistic was taken into account to analyze the correlation of the residual error values. For the presentation of homoscedasticity, ZRESID versus ZPRED graphs were utilized. Furthermore, a P-P plot of regression standardized residuals with a generated regression line and a histogram chart was used.

### 2.4. Participants

Two participants were observed in the presented study. The average male boxer mass (mean ± SD) was 79.25 KG ± 0.95 with an average boxer height of 177.5 ± 2.5 cm. The majority of the study was conducted utilizing material testing devices such as a Zwick/Roell material testing machine and developed validation devices such as the centrifugal apparatus.

## 3. Results

The statistical results of the validity of the predicted punch force determination using the developed sensor system compared to the determination of punch force by the Kistler force plate demonstrated a high linear regression between the two measurement methods.

The correlation analysis according to a Pearson product–moment correlation coefficient showed a high positive correlation of R = 0.995 between the force plate-derived force and sensor-derived force. A further analysis showed an adjusted R^2^ of 0.99 (Table 2) with a RMSE of 59.84 N.

The significance of the validation results is shown by means of an ANOVA. The performed F statistics showed a high significance of *p* < 0.001 with a confidence interval of 95% (F (1.123) = 11117.55, *p* < 0.001).

**Figure 4:**
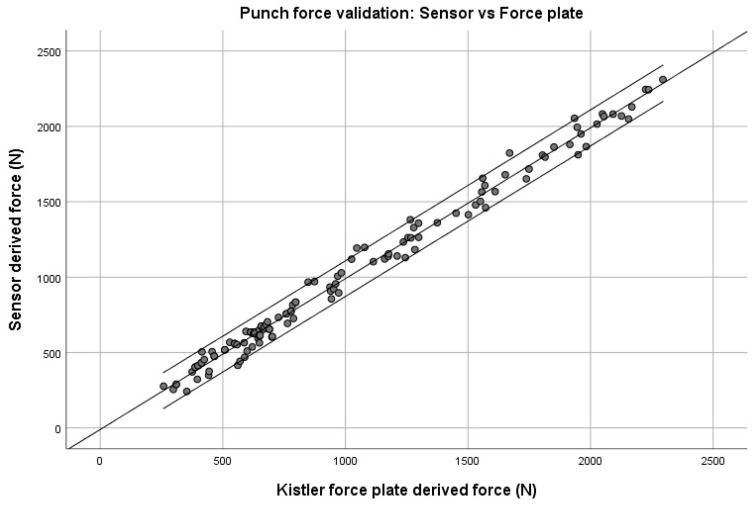
Punch force validation: Sensor-derived force (N) vs. Force plate-derived force (N).

Figure 4 presents the validation results for the peak punch forces determined by the developed boxing monitoring system compared to the Kistler force plate-determined peak punch forces with a displayed alpha of 5% confidence interval of the linear regression model, for a total of 125 punches thrown with a punch force ranging from 242 N up to 2310 N.

**Figure 5:**
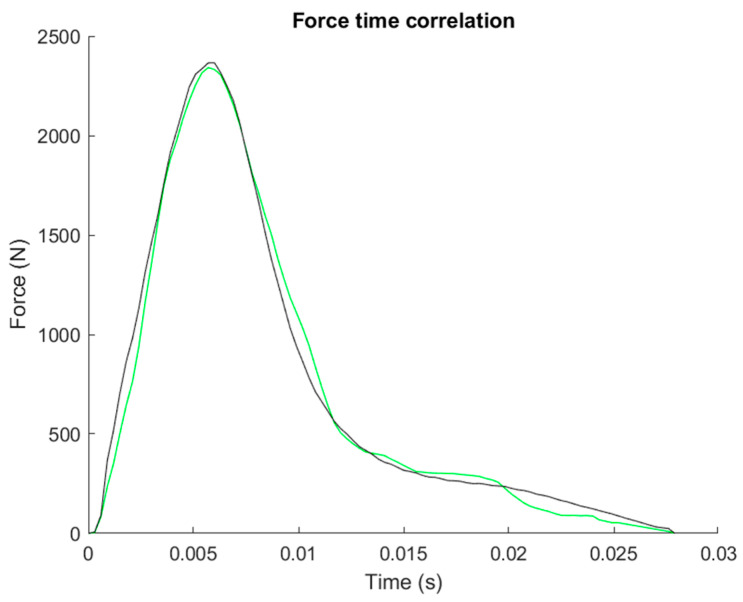
Force-time progression sensor (green) vs. Kistler force plate (black).

To further analyze the impact force, the time force progression of the punch force determined with the sensor (green) was analyzed with respect to the force time progression measured by the force plate (black). This analysis shows a correlation of the force–time curves of R = 0.98. The sensor-derived force-time curve shows a symmetrical leptokurtic curve pattern by comparison with the Kistler force plate-derived force-time profile in Figure 5.

The statistical analysis of the impact acceleration using the built-in inertial sensors, when compared to the acceleration of the validation centrifuge, shows a high linear regression between the two measuring methods used. The correlation analysis according to Pearson shows a correlation of R = 1.0 (adjusted R^2^ = 1.0) for the *x*-axis, a R = 1.0 (adjusted R^2^ = 1.0) for the *y*-axis and a Pearson R = 1.0 (adjusted R^2^ = 1.0) for the *z*-axis acceleration. The acceleration range tested ranged from −200 g to +200 g, determined by the inertial sensor and the acceleration measured by the centrifugal validation device.

The results of the F statistics show a significance of the measurement results of *p* < 0.001 with an applied confidence interval of 95% for all three axes.

For the *x*-axis, a maximum standard deviation of ±1.48 g at a centrifugal acceleration of −170 g and a maximum root mean square error of 3.21 at −200 g was observed. A maximum standard deviation of ±1.51 g was measured for the *y*-axis at a centrifugal acceleration of −120 g and a maximum root mean square error of 3.54 at +200 g. The maximum standard deviation for the *z*-axis was determined at ±0.83 g at a centrifugal acceleration of 80 g and a maximum root mean square error of 1.58 at +200 g.

The results of the regression analysis for the yaw, pitch and roll angle shows that the sensor model shows a Pearson R = 0.99 of sensor-derived angular rotation around the yaw axes. The analyses of the pitch axes resulted in a Pearson R = 0.99 and an R = 0.99 for the roll axes. Significance was analyzed with *p* < 0.001 for the applied F statistic, using an alpha of 5%.

The measurement accuracies achieved using the developed validation devices could be confirmed in a further validation examination of the sensor output data in comparison to the Vicon motion capture system. The validation of the sensor data was obtained during a test subject survey with a correlation to the Vicon measurement system with a Pearson R = 0.98 (*p* < 0.001) of the measured sensor accelerations and an R of 0.98 (*p* < 0.001) of the measured angular rotation for the three examined axes of rotation and accelerations with an alpha of 5%.

The system-derived velocity determination based on the developed zero-velocity-update was also validated using a marker-based Vicon motion capture system. The validation of the system-derived punch velocity showed a high significant correlation compared to the Vicon motion capture-derived punch velocity with R = 0.97 (*p* < 0.001) with a level of significance of 5%. The validation of the automatic determination of punch-time showed a significant correlation with the Vicon motion capture system of R = 0.95 (*p* < 0.001).

## 4. Discussion

The developed sensor system will be used in the applied field of boxing training, sparring and competition for athlete performance monitoring purposes. The knowledge gained from experimental data can offer coaches and athletes themselves a tool for analyzing the requirements of a specific punching movement pattern when compared to the movement pattern of an experienced athlete, with the help of the developed boxing monitoring system. The findings can further be used to apply technology analysis for talent identification and promotion in combat sports by the system as it is presented in this paper. Coaches and performance centers can, thus, benefit from this measurement system, as the technical performance of boxing strokes can be measured, and technique correction can be made in the interests of the athlete by objective data. Therefore, this experimental study was conducted to perform a quantitative analysis to validate the novel developed measurement system.

The impact force validation refers solely to the gold standard of a Kistler force plate, whereas the inertial measurement unit validation is based on generally accepted but non-standardized methods, such as the specially developed centrifugal device for acceleration validation, potentiometer instrumented gimbal device for angular rotation and an overall Vicon motion capture analysis for the validation of fist rotation, trajectory and punch velocity.

The first part of the study validates the newly developed pressure measurement system with a force measurement platform.

Each system was sampled at different frequencies. Therefore, a data interpolation was conducted with the developed sensor system at 1000 Hz and the Kistler force plate with a frequency of 10,000 Hz. This is due to the processing limitations of the microcontroller used to control the boxing monitoring system. Furthermore, the developed monitoring system has an inbuild threshold of 200 N. This inbuilt threshold is programmed to extract noise from so-called pit pat punches from the data analyses.

The designed, calibrated and incorporated pressure sensor results demonstrate its great applicability for boxing bouts. The presented statistical analysis of the punch force determination validation shows that with an R^2^ of 0.99 the developed sensor system enables a significant determination of the impact force while punching compared to the gold standard of a Kistler force plate. The sensor system revealed even greater accuracy for punch forces above 1000 N. Whereas the punch force determination accuracy was reduced to a R^2^ of 0.94 for forces below 1000 N. The observed accuracy of R^2^ = 0.94 was nevertheless within the acceptable accuracy range defined at the beginning of the research work for the dynamic testing.

The analyses of the force–time curve of the monitoring system shows an identical pattern in comparison to the Kistler force plate-derived force–time curve (Figure 5).

During the validation study, the developed sensor system allowed for detecting every punch that was applied to the force plate, and thus, to identify and count all the applied blows from 242 N to 2310 N with a 100% detection accuracy without sensor interferences.

The accuracy for all three axes of the accelerometer, gyroscope and magnetometer of the inertial measurement system is also of significant importance. These parameters are used to distinguish between punching technique for subsequent biomechanical analyses experiments on different punching techniques and their effects. Therefore, the validation was conducted in consecutive steps.

The validation of the acceleration was carried out in a first test by means of drop testing. Although these tests showed a high accuracy of R^2^ = 0.99, this type of validation method is limited. First, the accuracy cannot be correlated with a reference input value and second, the validation range is limited to ±1 g of the Earth’s gravitational acceleration. For the validation of the entire sensory measuring range of ±200 g, a special validation centrifuge with sensor mount was subsequently developed and manufactured [26,27,28]. To simplify the validation of the acceleration, a sensor mount with a 45° inclination was mounted on the rotation platform in order to measure the linearity over two axes simultaneously. During the stepwise testing, it became apparent, that due to the higher rotational speeds of the centrifuge required for this method, that the driving motor, at a simulated rotational acceleration of 180 g did not allow any further acceleration as an internal overload control led to the motor being switched off. To validate the entire measuring range of the sensor, the three axes were then individually aligned and validated in a flat position on the rotation platform perpendicular to the center of rotation to avoid the problem of internal overload control of the driving motor. With the help of this change of the sensor positioning, the simulated measuring range could be extended up to 200 g. The problem of the internal emergency stop was registered again after a constant acceleration for approximately two seconds at an acceleration of ±200 g. Due to this problem, the acceleration of 200 g could only be measured for a limited instant, unlike the previous measuring stages. Since the ideal measuring range of the sensor is within a range of 10% to 90% of the sensor and accelerations of ±200 g are unlikely, this limitation can be disregarded and was, therefore, neglected in the further validation process. A stronger motor would eliminate this problem, but a new drive shaft mount would need to be designed and manufactured for this purpose. Due to the negligible limitation and the possibility of measuring an acceleration of ±200 g for at least two seconds, this limitation has finally been ignored.

For further analysis, the sensor-derived acceleration was validated through long-term tests. For the validation period, a maximum period of 45 min was chosen to cover the maximum fight duration that can be achieved in a 12-round fight, as is common in the sport of boxing. A maximum deviation of 0.02 g was measured over a test interval of 5, 15 and 45 min. This small deviation was neglected in the further course of the experimental studies.

The final comprehensive validation of the angular rotation and velocity during the execution of boxing punches showed a high significance compared to the Vicon motion capture system, and thus, demonstrates the developed sensor system’s accuracy and reliability for the use in further experimental studies in laboratory as well as non-laboratory experimental settings.

## 5. Conclusions

Given the results of the punch force determination, as well as the measurement of acceleration and movement determination in three-dimensional space, the outcome of the validation experiments conducted demonstrate the significant accuracy of the measurements in predicting boxing-specific biomechanical movement parameters while punching (Table 3).

According to the validation results, the use of piezoresistive pressure sensors with the application of a dedicated calibration and filter method, enables the measurement of impact forces and motion kinetics in the field of combat sports, with the aid of comparatively inexpensive sensors of significantly great accuracies compared to a Kistler force plate and Vicon motion capture system. The rotation in a three-dimensional space shows furthermore, the possibility to replace a camera system to some extent to be able to display the hand trajectory and punch acceleration in three dimensions. The sport of boxing and other combat disciplines have specific movement patterns which are not analyzed during competition. The developed monitoring system makes it possible to investigate these impact movements in the field and to determine the impact effectiveness from the obtained and analyzed information.

Furthermore, the experiment outlines the critical importance of the validation process for new and unique monitoring systems. The most important criterion for developed sensor systems from and especially for scientific applications is the accuracy of the data acquisition method. These sensor systems have a fundamental influence on scientific research results and in this respect on the derived insights of the information provided by the measurement system used.

The acquired information based on the comprehensive methods stated for the sensor validation is of fundamental importance for the application and execution of upcoming field studies, to expand the biomechanical scientific understanding of the sport of boxing.

## Figures and Tables

**Table 1:** Dynamometry in the punching force literature modified according to Lenetsky [12] (p. 2) in chronological order.

Study	Subjects	Force Measuring Equipment	Punches Tested
Joch et al., (1981) [20]	Elite (n = 24), national-level (n = 23), intermediate-level boxers (n = 23)	Water filled punching bag equipped with pressure sensors	Straight punch
Atha et al., (1985) [10]	Professional heavy weight boxer (n = 1)	Padded pendulum equipped with piezoelectric force transducer	Cross Punch
Smith et al., (2000) [14]	Elite (n = 7), intermediate (n = 8), and novice (n = 8) boxers	Wall-mounted force plate (4 triaxial piezoelectric force transducers) with a boxing manikin cover	Rear and front hand straight punch
Birken et al., (2001) [21]	Professional Boxer (n = 2)	Boxing glove and punching bag equipped with accelerometer	Cross punch
Dyson et al., (2005) [22]	Male competitive amateur boxers (n = 6)	Boxing dynamometer manikin which was matched to the shoulder height of each subject	Singular and combination straight punches in a prescribed sequence. Rear and lead hand.
Walilko et al., (2005) [15]	Olympic boxers weighing from 48 to 109 kg (n = 7)	Hybrid III dummy equipped with a 6-axis load cell in the neck, a Tekscan’s pressure sensor in the dummy’s face, and Endevco accelerometers on the boxer’s hands	Straight Punch
Chadli et al., (2014) [23]	Amateur college athletes (n = 11)	Torsion bar fixed on a frame consisting of strain gauge sensors and two accelerometers. One attached to the target, and one worn inside the glove	Strike with maximum power
Loturco et al., (2016) [17]	Amateur boxers from Brazilian National Team (n = 15)	Force platform covered by a body shield was mounted on a wall at height of 1 m perpendicular to the floor	Jab and cross punch

**Table 2:** Regression analysis summary for the validation of sensor-derived punch force determination.

Variable	B	95% CI [Llower Limit–Upper Limit]	ß	t	*p*
Constant	−10.164	[−32.05–11.72]		−0.92	0.36
Kistler Force Plate	1.00	[0.98–1.02]	0.99	105.44	0.00

Note. R^2^ adjusted = 0.99. CI = Confidence interval for B.

**Table 3:** Validation results.

Validation Method (Sensor System vs. …)	Variable	R	*p*
Gimbal device	Angular rotation (°)	0.99	<0.001
Centrifugal device	Acceleration (g)	1.0	<0.001
Kistler Force Plate	Punch Force (N)	0.99	<0.001
	Force-time progression	0.98	<0.001
Vicon Motion Capture System	Angular rotation (°)	0.98	<0.001
	Acceleration (g)	0.98	<0.001
	Velocity (m/s)	0.97	<0.001
	Punch time (throw, contact and retraction phase) (ms)	0.95	<0.001

Note. A 95% Confidence Interval was applied.

## Data Availability

The data presented in this study are available upon request from the corresponding author. The data are not publicly available due to further research work. Data will be made available in the near future.
